# THE U-SHAPED RELATIONSHIP BETWEEN DAILY ENERGY INTAKE AND ALL-CAUSE MORTALITY IN US ADULTS

**DOI:** 10.1093/geroni/igad104.1615

**Published:** 2023-12-21

**Authors:** Samaneh Farsijani, Ziling Mao, Anne Newman

**Affiliations:** University of Pittsburgh, Pittsburgh, Pennsylvania, United States; University of Pittsburgh, Pittsburgh, Pennsylvania, United States; University, Pittburgh, Pennsylvania, United States

## Abstract

The association between low energy intake and longevity observed in non-human studies has not been confirmed in large-scale human investigations. Therefore, we determined the associations between daily energy intake and all-cause mortality in US adults. We included 33,094 US adults (age>19 years) from the National Health and Nutrition Examination Survey (2003-2018). Total energy intake was obtained from 24-hour food recalls and mortality was determined through December 2019 using the National Death Index. Survey-weighted multivariable Cox proportional hazard models were used to calculate all-cause hazard ratios (HR) across categories of energy intake. HR models were stratified by age, sex, race, and BMI, while controlling for potential covariates. During a median follow-up of 96 months, 4,162 participants died. Low energy intake (< 1,000 kcal/day) was associated with higher mortality (HR=1.53 [95% CI 1.26-1.86]) vs. the reference category (2,000-2,500 kcal/d) independent of age, sex, race, BMI, and income. Similarly, low energy intake was associated with higher mortality risk (P< 0.05) in middle-aged (40-64y) and older (≥65y) adults, men, women, and Whites. We observed the same negative associations between energy intake and mortality in normal-weight (18.5-24.9 kg/m2), over-weight (25.0-29.9), and obese (≥30.0) individuals (P< 0.05). The only exception was in Blacks, where lower energy intake was associated with lower mortality (HR=0.75 [0.57-0.99]). Higher energy intake (≥4,000 kcal/d) was also associated with higher mortality, though HRs were not significant, except in young (20-39y), women, and normal weight adults. Our result showed a U-shaped relationship between total energy intake and mortality, like the observed relationship between BMI and mortality.

